# A signature constructed with mitophagy-related genes to predict the prognosis and therapy response for breast cancer

**DOI:** 10.18632/aging.204209

**Published:** 2022-08-05

**Authors:** Yingan Zhao, Yingjue Zhang, Chen Dai, Kai Hong, Yangyang Guo

**Affiliations:** 1 Department of Hepatobiliary Surgery, Ningbo First Hospital, Ningbo 315010, Zhejiang, China; 2 Department of Molecular Pathology, Division of Health Sciences, Graduate School of Medicine, Osaka University, Suita, Osaka 565-0871, Japan; 3 Medicine School, Ningbo University, Ningbo 315211, Zhejiang, China

**Keywords:** breast cancer, biomarker, prognosis, treatment

## Abstract

Over the past decades, the incidence and mortality rates of breast cancer (BC) have increased rapidly; however, molecular biomarkers that can reliably detect BC are yet to be discovered. Our study aimed to identify a novel signature that can predict the prognosis of patients with BC. Data from the TCGA-BRCA cohort were analyzed using univariate Cox regression analysis, and least absolute shrinkage and selection operator (LASSO) analysis was performed to build a stable prognostic model. Subsequently, Kaplan–Meier (K–M) and receiver operating characteristic (ROC) analyses were performed to demonstrate the predictive power of our gene signature. Each patient was assigned to either a low- or high-risk group. Patients with high-risk BC had poorer survival than those with low-risk BC. Cox regression analysis suggested that our signature was an independent prognostic factor. Additionally, decision curve analysis and calibration accurately predicted the capacity of our nomogram. Thus, based on the differentially expressed genes (DEGs) of mitophagy-related tumor classification, we established a 13-gene signature and robust nomogram for predicting BC prognosis, which can be beneficial for the diagnosis and treatment of BC.

## INTRODUCTION

Breast cancer (BC) is one kind of malignant tumor and accounts for one-quarter of cases of cancer in women. Over the past decades, the incidence and mortality of BC in developing countries have increased rapidly, especially in China [1, 2]. Approximately 2 million people were newly diagnosed with BC and 600 thousand died of this malignant tumor worldwide in 2018 [3]. There are many known risk factors involved in the tumorigenesis and progression of BC, such as obesity, genetic factors, family history, and endocrine factors. According to specific protein expressions such as human epidermal growth factor receptor 2 (HER2), estrogen receptor (ER), and progesterone receptor (PR), four subtypes of BC were identified: HER2-enriched (HER2+), TNBC (ER−, PR−, HER2−, triple negative breast cancer), luminal A (ER+ or PR+, HER2−), and luminal B (ER+ or PR+, HER2+) [4, 5]. Despite advances in surgical treatment, endocrine therapy, radiation treatment, chemotherapy and targeted therapy, the five-year survival rate of BC, especially in TNBC, still low owing to distant metastasis [6, 7]. Although there have been many studies on BC, the mechanisms underlying its progression remain unclear. Thus, building a novel gene signature and clarifying the potential mechanisms in BC patients are critically needed.

Mitophagy is a selective process in which mitochondria are selectively cleared through the autophagic pathway. Mitophagy is critical for cellular homeostasis, and cells can eliminate dysfunctional mitochondria or reduce mitochondrial numbers via the mitophagy mechanism [8, 9]. Mitochondria are important cellular organelles that perform many different functions, from cell death regulation and energy generation to immune responses and fatty acid oxidation [10, 11]. Mitophagy can be mediated by multiple molecular mechanisms, such as the NIX, FundC1, and PINK1/Parkin signaling pathways. Mitophagy disorders are closely related to various cancers, including rectal cancer, lung cancer, and BC. Deng et al. revealed that degradation of ULK1 attenuates mitophagy and promotes BC bone metastasis [12]. Although there have been many studies on mitophagy, its role in BC has not been fully studied.

In our analysis, we established a stable signature, including 13 genes, based on differentially expressed genes (DEGs) in mitophagy-related tumor classification. Kaplan–Meier (K–M) and evaluation analyses of the signature were performed across the TCGA-BRCA project. The signature was validated in the independent BC cohort GSE20685. In addition, tumor microenvironment (TME), immunotherapy response, drug sensitivity, and putative molecular pathways were investigated. Taken together, these results provide a novel treatment option and predictive tool for BC.

## MATERIALS AND METHODS

### Data

Expression data and clinical information of BC samples were downloaded from the Gene Expression Omnibus (GEO) (GSE20685, https://www.ncbi.nlm.nih.gov/geo/) database and The Cancer Genome Atlas (TCGA) (https://portal.gdc.cancer.gov/repository). The TCGA-BRCA project was used for breast cancer analysis. The batch effect of the GEO data was eliminated by normalization. Transcriptome profiling was converted into fragments per kilobase million (FPKM) and combined with clinical information for further analysis. In addition, we obtained 29 mitophagy-related genes (MRGs) from the Pathway Unification online database (Supplementary Table 1).

### Identification of differentially expressed MRGs

To explore the expression profile of MRGs in breast cancer, limma algorithm was used to identify the differentially expressed MRGs by “limma” R package across the TCGA-BRCA dataset [13]. DEGs were screened using the criteria FDR < 0.05. A protein–protein interaction (PPI) network was used to determine the interaction of MRGs using the Search Tool for the Retrieval of Interacting Genes (STRING, https://string-db.org/) [14]. Additionally, a correlation network of m7G-related DEGs was formed (interaction score cutoff = 0.2) using the “reshape2” and “igraph” packages in R [15].

### Consensus clustering

Distinct consensus clustering was conducted using differentially expressed MRGs. The threshold was set as iteration = 100 and the resample rate = 80%. Consensus clustering analysis was performed using the “ConsensusClusterPlus” R package and the survival difference between different clusters was evaluated using the “survival” R package [16]. Differences in clinical characteristics between each cluster were shown by a heatmap across the TCGA-STAD project using the “pheatmap” R package.

### Development of a gene prognostic signature

First, we analyzed DEGs between BC subtypes using the criteria FDR < 0.05. Prognostic differentially expressed genes were identified using univariate Cox analysis. Next, we used the R package “glmnet” to perform LASSO Cox regression analysis of these prognostic DEGs [17]. The risk score of each BC was calculated as follows: $\text{Risk score}=\sum_{i=1}^{n}Coef(i)\times Expr(i),$ and BC samples were assigned to two subgroups according to the median risk score [18]. Sequentially, difference of survival of low- and high-risk BC was analyzed in TCGA-BRCA as the training dataset and GSE20685 as the test dataset, using the “survival” R package. The area under the receiver operating characteristic (ROC) curve (AUC) values were used to establish the prognostic value of the MRG signature across TCGA-BRCA and GSE20685 [19]. Besides, risk score distribution and the survival status were visualized by the “pheatmap” R package across TCGA-BRCA and GSE20685. Principal component analysis (PCA) and t-distributed stochastic neighbor embedding (t-SNE) analysis were used to evaluate the ability of the signature to distinguish low- and high-risk BC across TCGA-BRCA and GSE20685 [20, 21].

### Prognostic values of the signature and subgroup analysis

Multivariate and univariate Cox regression analyses of the signature and several clinical characteristics were used to identify the independent prognostic factors. Moreover, the expression profiles of genes contained in the signature, as well as the correlation between the signature and clinical characteristics, are presented in a heatmap. Additionally, differences in risk between distinct clinical subgroups were evaluated using the limma algorithm, and the survival of low- and high-risk BC in distinct clinical subgroups was assessed using K–M analysis [22].

### Construction and verification of a nomogram

A nomogram was constructed with the stable signature and several clinical characteristics using the “rms” and “regplot” R packages [23]. A calibration curve was constructed to determine the predictive probability of the nomograms. ROC and decision curve analysis (DCA) analyses were performed to demonstrate the robustness of the nomogram as a predictive factor [24].

### Functional enrichment analyses

Gene Ontology (GO) analysis, including BP, CC, and MF analyses, was conducted to evaluate the putative cellular functions of DEGs in low- and high-risk BC [25]. Kyoto Encyclopedia of Genes and Genomes (KEGG) analysis was performed to identify the relevant pathways related to DEGs in low- and high-risk BC [26]. The top five enriched pathways of low- and high-risk BC were visualized through gene set enrichment analysis (GSEA) analysis, and enriched pathways of low- and high-risk BC were assessed by gene set variation analysis (GSVA) analysis [27, 28]. The functional enrichment analysis was conducted by “limma,” “org.Hs.eg.db,” “clusterProfiler,” “enrichplot,” “ggplot2,” “GOplot,” and “GSVA” R packages [29].

### Tumor immune cell infiltration

We established the immune cell infiltration patterns of low- and high-risk BC using the “TIMER,” “CIBERSORT,” “CIVERSORT-ABS,” “QUANTISEQ,” “MCPCOUNTER,” “XCELL,” and “EPIC” algorithms, visualized with a heatmap. In addition, scores of infiltrating immune cells (CD4+T cells, aDC, B cells, DC, iDC, mast cells, CD8+T cells, NK cells, neutrophils, pDC, macrophages, T helper cells, Tfh, Th1, Th2, Treg, etc.) and immune functions (APC-co-inhibition, APC-co-stimulation, CCR, check-point, cytolytic-activity, and et al.) of low- and high-risk BC were also evaluated across TCGA-BRCA and GSE20685 datasets [30].

### Drug sensitivity analysis

Studies have demonstrated that higher inhibitory concentration (IC50) values are related to lower antitumor capacity. To investigate drug sensitivity, we used our established model in the genomics of drug sensitivity in cancer (GDSC) (https://www.cancerrxgene.org/). The R package “pRRophetic” was used to analyze drug sensitivity [31].

### Statistical analysis

R software (version 4.1.3) and Perl-5.32 were applied for statistical analysis. Subgroup comparisons were conducted using the Wilcoxon test or Student’s *t*-test. Spearman’s correlation analysis was performed to analyze the correlation between the two continuous variables. The Kruskal-Wallis test was used to compare the three groups. Statistical significance was defined as *p* < 0.05.

## RESULTS

### Identification of DEGs between tumor and normal samples

**Figure 1:**
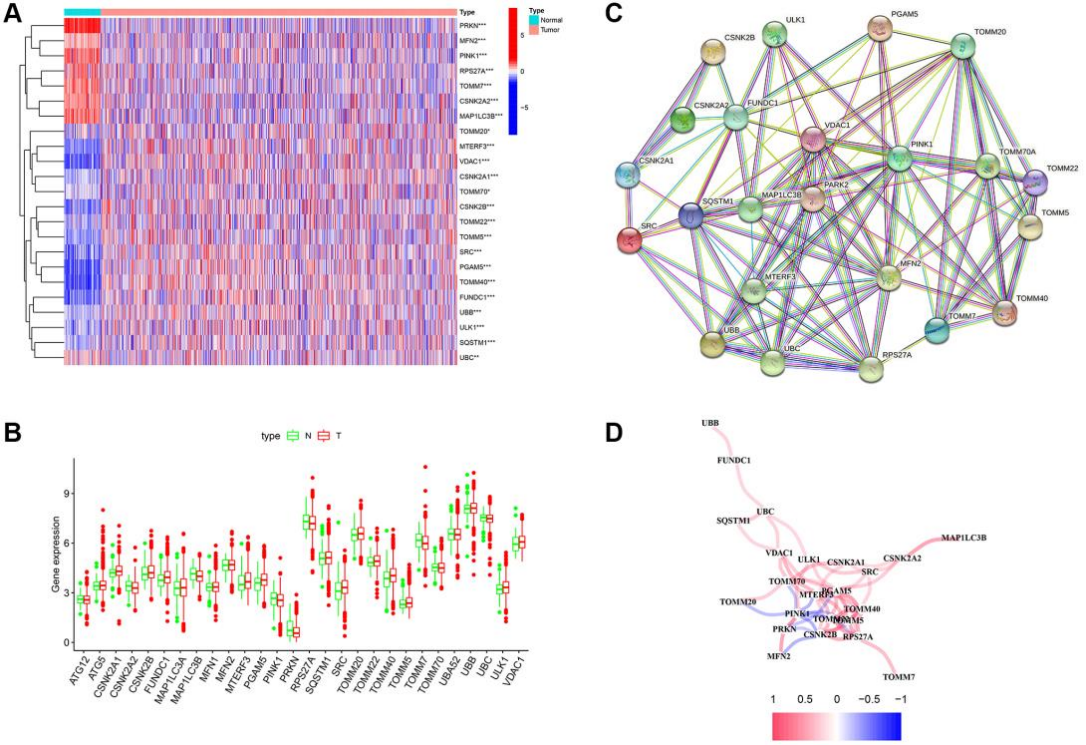
**Differentially expressed genes (DEGs) related to mitophagy were identified between cancer and normal tissue.** (**A**) Heatmap of mitophagy-related genes (MRGs) expression profiles. (**B**) Boxplots of the expression of DEGs. (**C**) Protein-protein interaction (PPI) network of DEGs. (**D**) Correlation network of DEGs. Red represents positive correlations while blue represents negative correlations. ^*^p < 0.05; ^**^p < 0.01; ^***^p < 0.001.

The mRNA expression of 29 MRGs was evaluated between normal and tumor samples from TCGA database. Then, 23 DEGs were identified, 17 of which (CSNK2A1, CSNK2B, FUNDC1, MFN2, MTERF3, PGAM5, PRKN, SQSTM1, SRC, TOMM20, TOMM22, TOMM40, TOMM5, TOMM70, UBB, ULK1, and VDAC1) were upregulated, whereas six of these genes (CSNK2A2, MAP1LC3B, PINK1, RPS27A, TOMM7, and UBC) were downregulated (Figure 1A and 1B). In addition, PPI was used to explore the correlations between the MRGs (Figure 1C). The correlation network is shown in Figure 1D.

### Tumor classification based on MRGs

**Figure 2:**
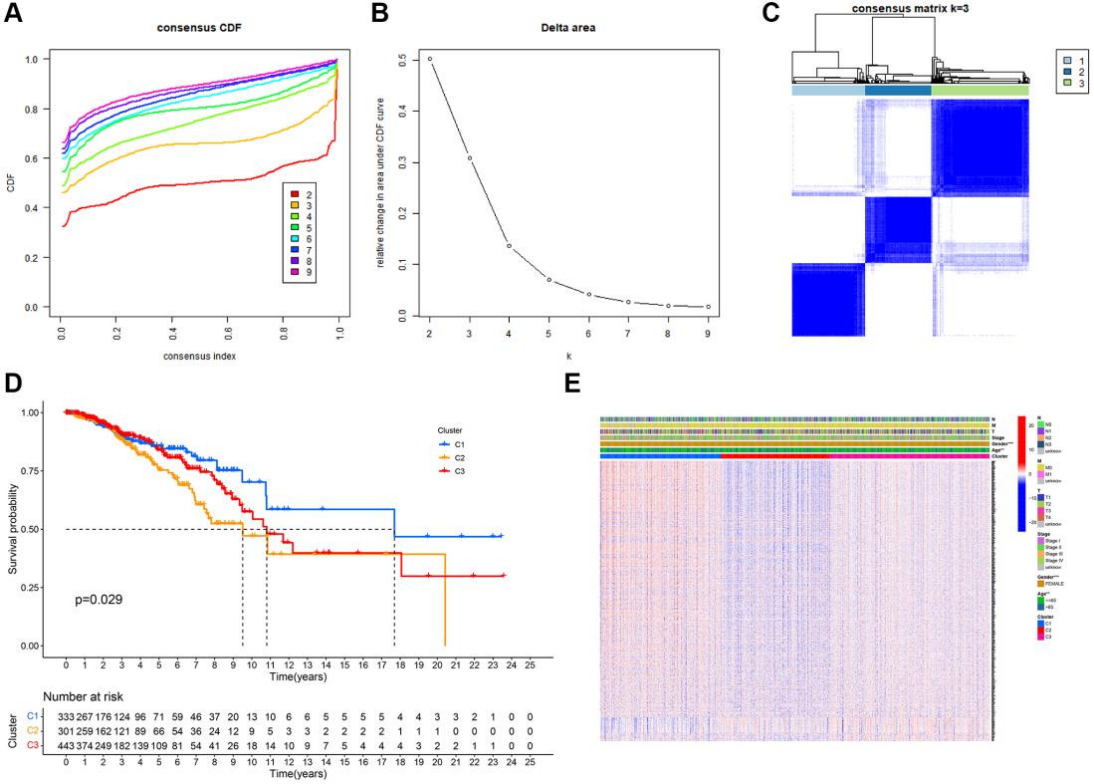
**Tumor classification based on mitophagy-related genes (MRGs).** (**A**) Cumulative distribution function (CDF) curves. (**B**) Delta area curve of consensus clustering. (**C**) Consensus clustering matrix. (**D**) Kaplan–Meier (K–M) survival analysis of the three subgroups. (**E**) Heatmap of DEG expression profiles in three subgroups.

Unsupervised clustering analysis was performed to evaluate the efficacy of MRGs on BC samples. According to the results of the relative change in the area under the curve (AUC) of the cumulative distribution function (CDF), the optimal cluster number was K = 3 (Figure 2A, 2B and 2C). BC samples were divided into three subtypes (*N* = 333, 301, and 443) based on mitophagy-related genes. The difference in survival among the three subtypes was significant (Figure 2D). DEGs between the three groups of subtypes were analyzed, and the heatmap combined with clinical information is shown in Figure 2E.

### Establishment of the 13-gene signature

**Figure 3:**
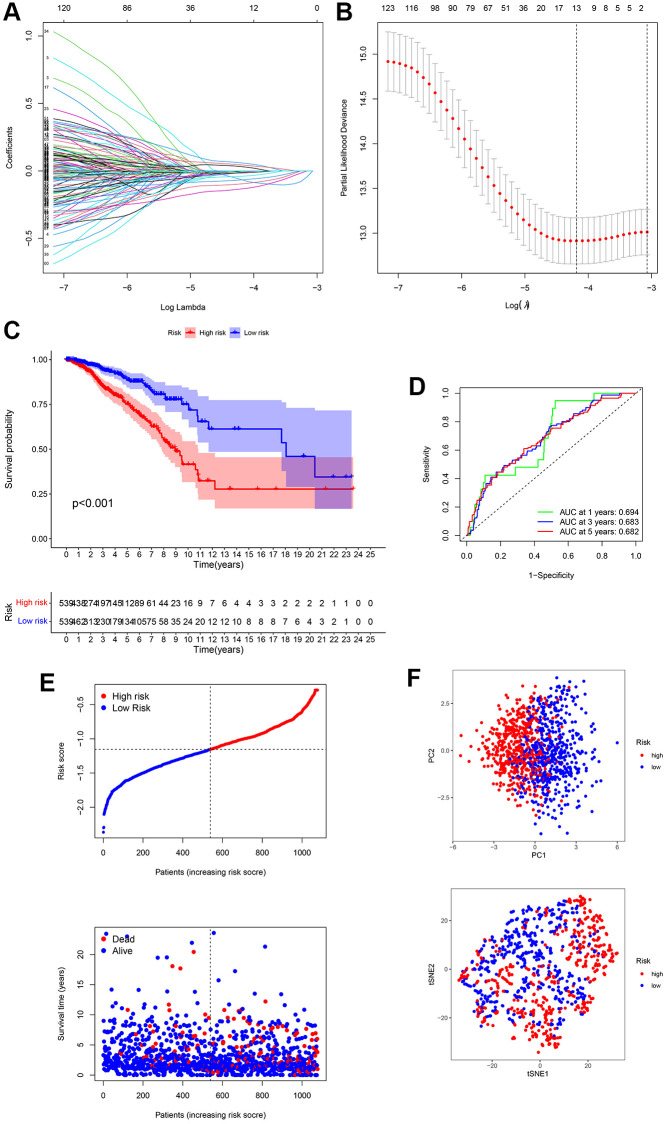
**Establishment of a 13-gene signature in TCGA cohort.** (**A**) Differentially expressed genes (DEGs) were penalized by LASSO Cox regression analysis. (**B**) Cross-validation of candidate genes based on the minimum lambda value. (**C**) Survival analysis between two risk subgroups. (**D**) Receiver operating characteristic (ROC) curve of the 13-gene signature. (**E**) Survival time and status of each breast cancer (BC) sample based on the risk score. (**F**) Principal component analysis (PCA) of the 13-gene signature.

DEGs between the three groups of subtypes were evaluated using univariate Cox analyses, and 13 genes were identified as prognosis-related across TCGA-BRCA. ADAM9, MAL2, and CLEC3A were risk genes (HR > 1), whereas TNFRSF14, RELB, SEMA3B, IGFALS, CEBPD, KRTCAP3, CCL19, CHAD, KRT5, and LTF were protective genes (HR < 1) (Figure 3A). We identified a 13-gene signature using the LASSO Cox regression analysis (Figure 3A and 3B). The formula for the risk score is as follows:

Risk score = (–0.065 × TNFRSF14exp.) + (–0.006 × RELBexp.) + (–0.132 × SEMA3Bexp.) + (0.059 × ADAM9exp.) + (–0.007 × IGFALSexp.) + (0.031 × MAL2exp.) + (–0.039 × CEBPDexp.) + (–0.095 × KRTCAP3exp.) + (–0.066 × CCL19exp.) + (–0.011 × CHADexp.) + (–0.014 × KRT5exp.) + (0.044 × CLEC3Aexp.) + (–0.005 × LTFexp.) (Figure 3A and 3B).

Patients with BC were divided into high- and low-risk groups based on the median value (Figure 3E). The survival curve suggested that high-risk BC patients had poorer survival rates than that of low-risk BC patients (Figure 3C). ROC analysis was performed to evaluate the predictive model constructed using the risk score. The AUC of the ROC curves at 1, 3, and 5-year were 0.694, 0.683, and 0.682, respectively (Figure 3D). In addition, PCA analysis presented that BC patients in different groups were well separated into different subtypes (Figure 3F).

### Validation of the risk signature

**Figure 4:**
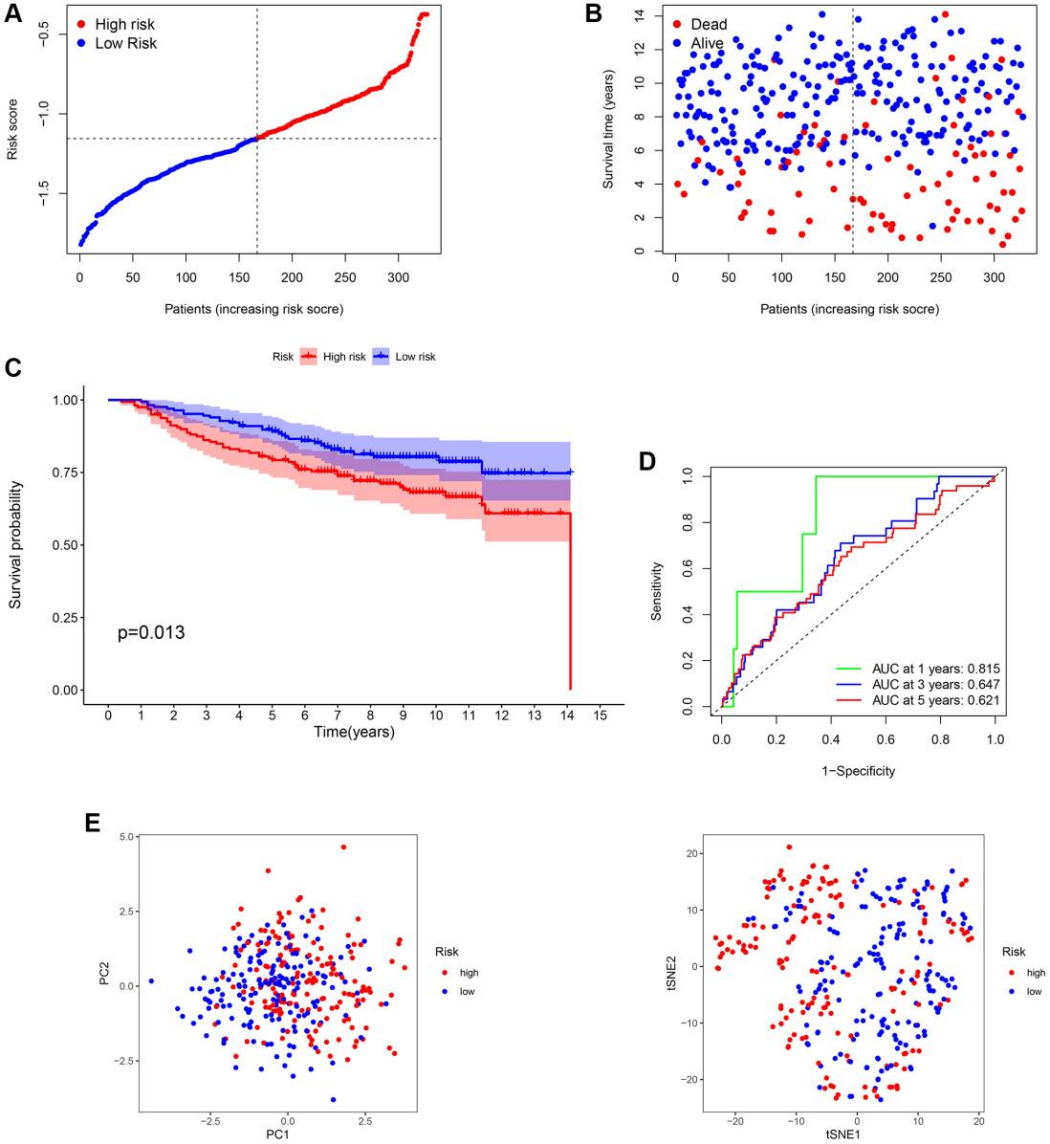
**Validation of the 13-gene signature in GSE20685.** (**A** and **B**) Survival time and status of each breast cancer (BC) sample based on the risk score. (**C**) Survival analysis between two risk subgroups. (**D**) Receiver operating characteristic (ROC) curve of the 13-gene signature. (**E**) Principal component analysis (PCA) of the 13-gene signature.

The GSE20685 dataset from the GEO database was selected for validation. First, mRNA expression levels were normalized for subsequent analysis. All the patients in the GEO cohort were divided into low- and high-risk subtypes (Figure 4A and 4B). Consistent with TCGA analysis, BC patients in the high-risk group had poorer survival rates (Figure 4C). The AUC of the ROC curve at 1-year, 3-year, and 5-year were 0.815, 0.647, and 0.621, respectively (Figure 4D). In addition, PCA displayed a moderate difference between the two groups (Figure 4E). The results of GSE20685 also demonstrated that our prognostic model had moderate predictive capability.

### Independent prognostic analysis of the risk model

**Figure 5:**
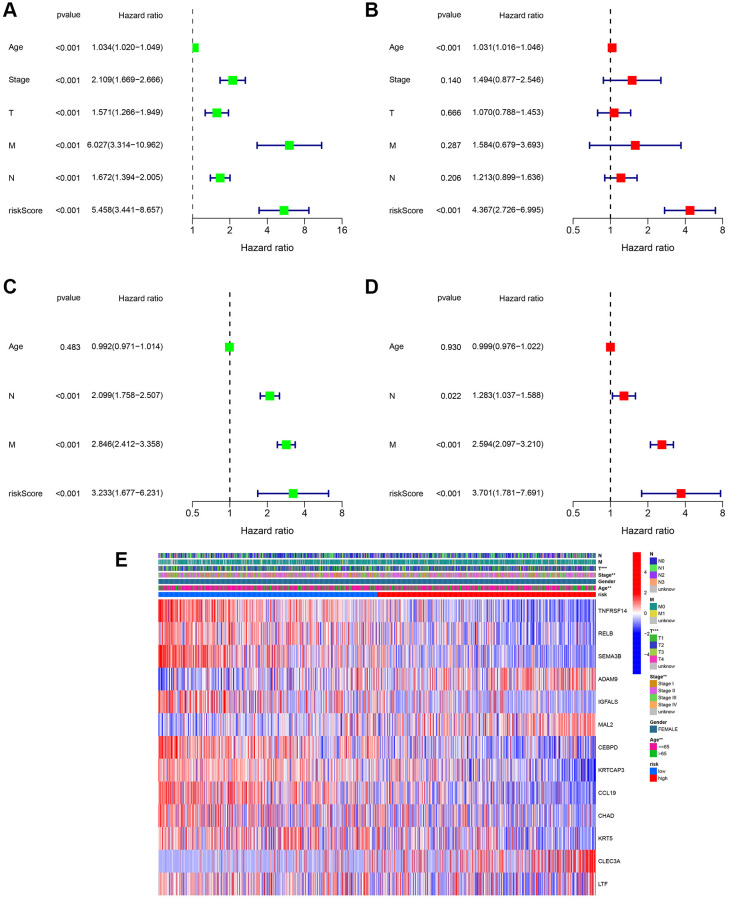
**Assessment of the clinical prognostic value of the risk score model in patients with breast cancer (BC) by univariate and multivariate Cox analysis.** (**A**) Univariate independent Cox analysis for TCGA cohort. (**B**) Multivariate independent Cox analysis for TCGA cohort. (**C**) Univariate independent Cox analysis for GSE20685. (**D**) Multivariate independent Cox analysis for GSE20685. (**E**) Heatmap of the 13-gene signature and clinicopathological manifestations.

Univariate and multivariate Cox regression analyses were performed to estimate independent prognostic factors for patients, univariate and multivariable Cox regression analysis was performed. The results revealed that the risk score (HR = 5.458, 95% CI = 3.441 – 8.657) was a prognostic factor in the TCGA cohort (Figure 5A). Multivariate analysis revealed that the risk score (HR = 4.367, 95% CI = 2.726 – 6.995) was an independent factor for BC patients (Figure 5B). Figure 5C shows that the risk score (HR = 3.233, 95% CI 1.677 – 6.231) was a prognostic factor in the GSE20685 dataset. Multivariate analysis revealed that the risk score (HR = 3.701, 95% CI = 1.781 – 7.691) was an independent factor for BC patients in the GSE20685 cohort (Figure 5D). Moreover, based on the TCGA cohort, a clinicopathological information heatmap was displayed, which showed that BC patients between the two groups showed a significant correlation with tumor stage, age, and T classification (Figure 5E).

**Figure 6:**
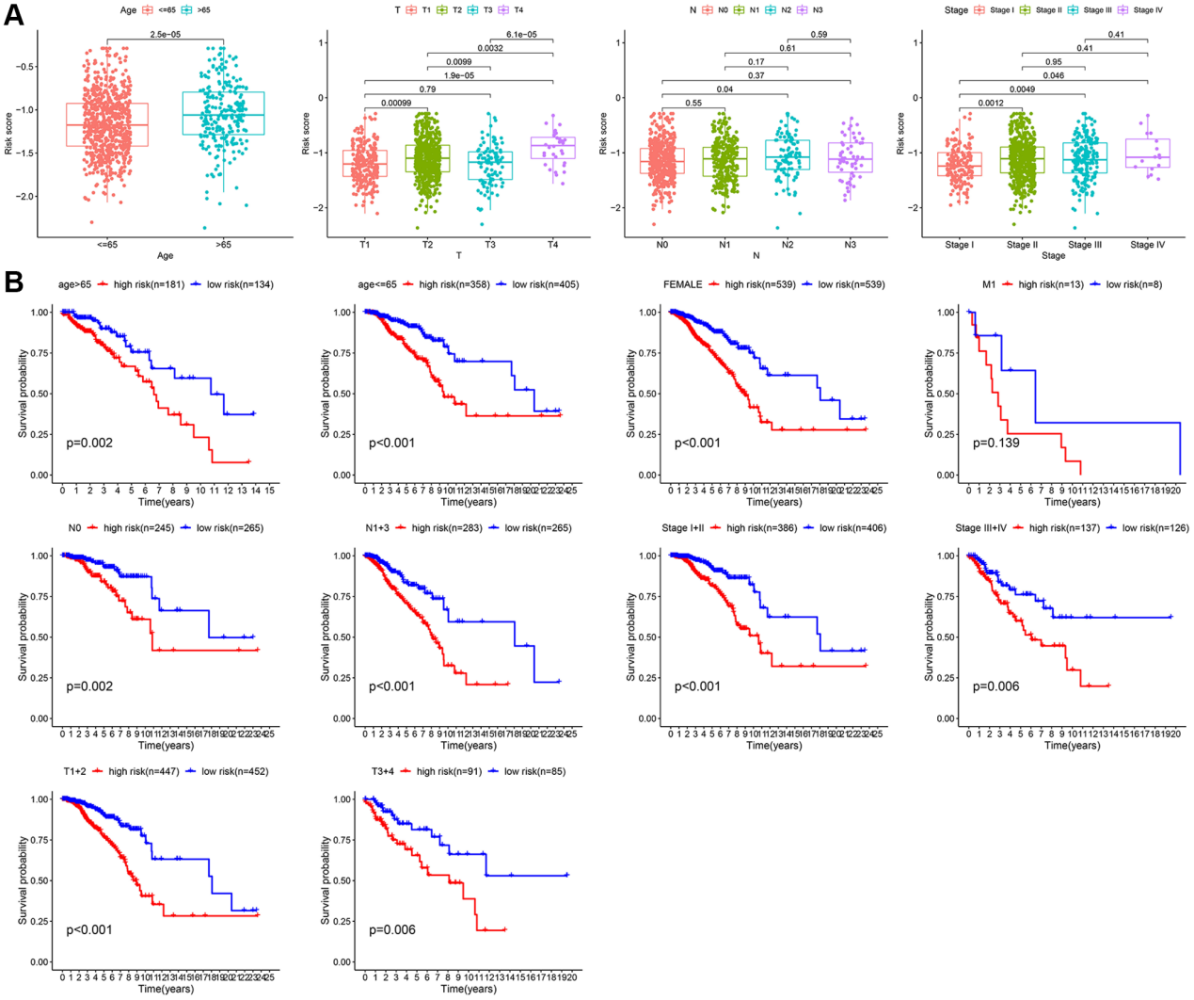
**Subgroup analysis of the risk score.** (**A**) Correlation of risk models with clinical characteristics. (**B**) Survival analysis between two risk subgroups during clinical subgroups.

Next, the correlation between the risk scores and clinical characteristics was investigated. As shown in Figure 6A, different subgroups, including N stage, age, T stage, and stage, had significantly different risk scores. To further verify the reliability of the risk model, subgroup analysis confirmed the differences in survival between the low- and high-risk groups in different cancer subgroups, including subgroups of age > 65 years, female sex, M1, age ≤ 65 years, N0, stage III-IV, N1-3, stage I-II, T1+2, and T3+4 (Figure 6B).

### Establishment of a prognostic nomogram for BC patients

**Figure 7:**
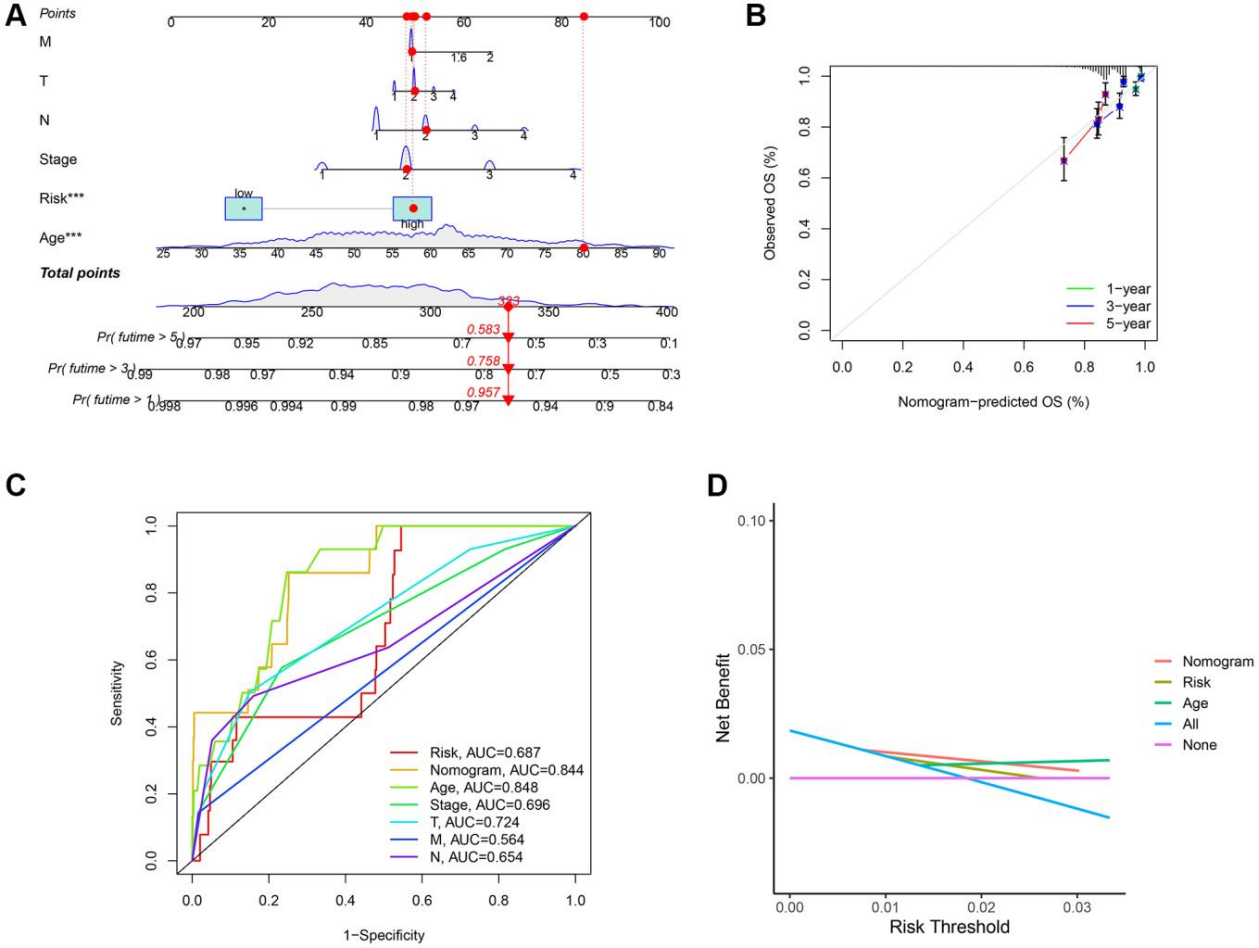
**Establishment of nomogram model and calibration curves.** (**A**) The predictive nomogram. (**B**) The calibration curves of the nomogram. (**C**) Receiver operating characteristic (ROC) curve analysis of the clinicopathological manifestations and nomogram. (**D**) The decision curve analyses (DCA) plot.

Based on TCGA cohort, we generated a new prognostic nomogram to predict BC patient survival (Figure 7A), which revealed that the prognostic nomogram could systematically predict the overall survival (OS) of BC patients at 1, 3, and 5 years. The calibration plots showed good agreement between the actual and predicted outcomes (Figure 7B). In addition, the AUC of the nomogram for predicting survival was 0.844 (Figure 7C), and DCA showed a robust predictive probability of the nomogram (Figure 7D).

### Functional enrichment of the risk signature

**Figure 8:**
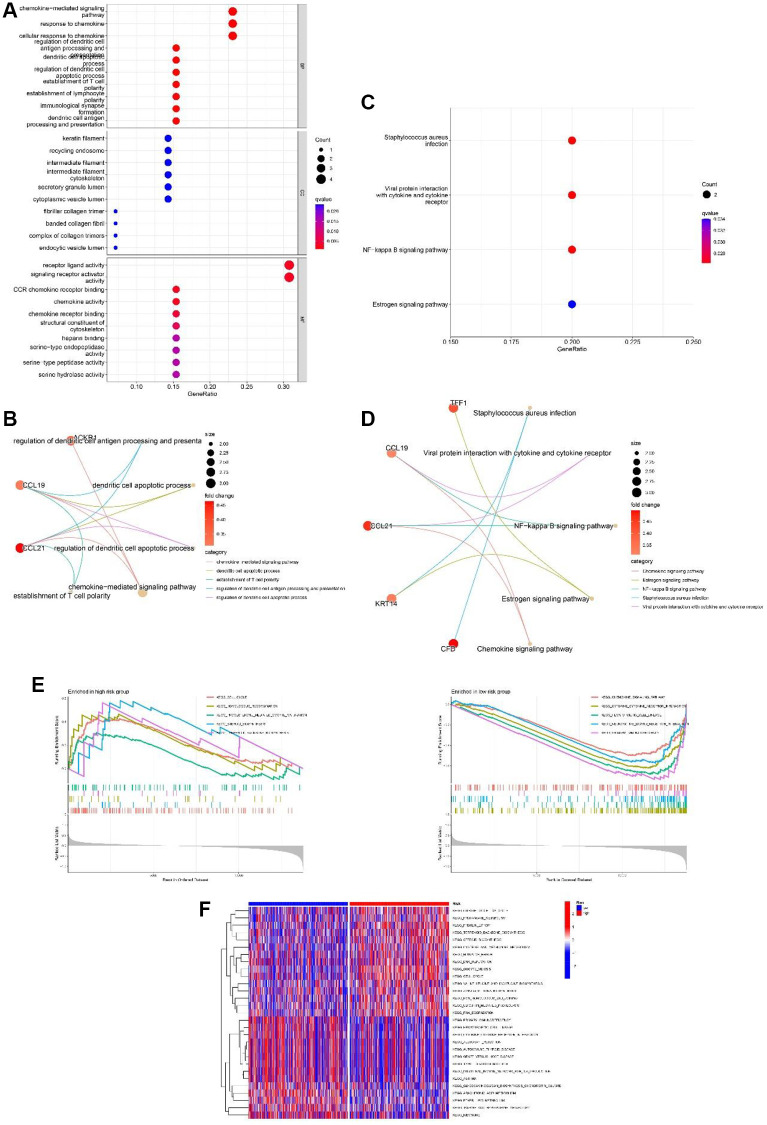
**Functional analyses of the 13-gene signature in the TCGA cohort.** (**A**, **B**) GO enrichment analysis of differentially expressed genes (DEGs) between the high and low-risk group. (**C**, **D**) KEGG enrichment analysis of DEGs between the high-risk group and low-risk group. (**E**) GSEA of high-risk group and low-risk group. (**F**) Gene set variation analysis (GSVA) of high-risk group and low-risk group.

To research the functional annotations of the 13-gene risk signature, we performed enrichment analysis on DEGs between the high- and low-risk groups. As shown in Figure 8A and 8C, GO enrichment revealed that these DEGs were mainly enriched in “response to chemokine” and “chemokine-mediated signaling pathway” chemokine-mediated signaling pathways. KEGG enrichment showed significant enrichment of “viral protein interaction with cytokine and cytokine receptor” and “NF-kappa B signaling pathway” (Figure 8B and 8D). GSEA was performed to evaluate the different pathways between the low- and high-risk groups. Results showed that “cell cycle,” “progesterone mediated oocyte maturation,” “homologous recombination,” “steroid biosynthesis” and “terpenoid backbone biosynthesis” were the top 5 enriched pathways in high-risk group. In the low-risk group, the top five enriched pathways were the “chemokine signaling pathway”, “hematopoietic cell lineage”, “cytokine-cytokine receptor interaction”, “neuroactive ligand receptor interaction, and “primary immunodeficiency” (Figure 8E). In addition, as shown in Figure 8F, from the heatmap of GSVA, significant differences in enriched functions between the low- and high-risk groups were observed.

### Comparison of the immune activity among subgroups

**Figure 9:**
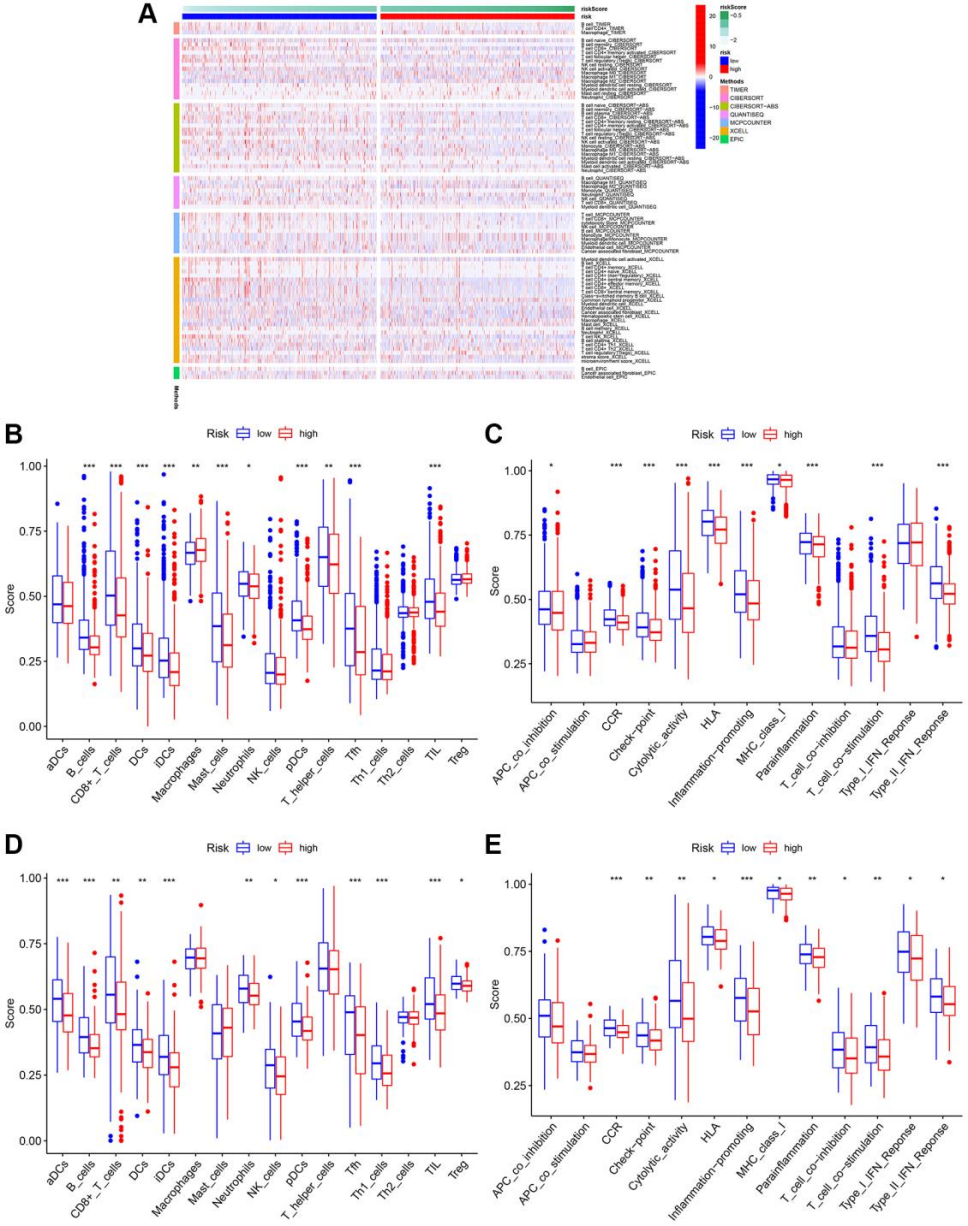
**Analysis of immune cell infiltration.** (**A**) Heatmap of immune cell infiltration. (**B**) Boxplot of immune cell infiltration in TCGA cohort. (**C**) Boxplot of immune function in TCGA cohort. (**D**) Boxplot of immune cell infiltration in GSE20685. (**E**) Boxplot of immune function in GSE20685.

Previous studies have shown that TME plays a significant role in tumor development [32, 33]. To investigate the differences in immune-related annotations and immune cell infiltration between subtypes, ssGSEA was performed. As shown in Figure 9A, we assessed the differences in immune cell infiltration between subtypes using seven different algorithms. aDCs, NK cells, Tregs, Th2 cells, and Th1 cells did not differ between the two groups in the TCGA cohort. B cells, DC, iDC, CD4+T cells, mast cells, CD8+T cells, neutrophils, pDC, T helper cells, Tfh, and TIL infiltrated at a greater rate in the low-risk subgroup, while macrophages infiltrated at a greater rate in the high-risk group (Figure 9B). CCR, cytolytic activity, checkpoint, HLA, Type II IFN response, MHC class I, parainflammation, T cell co-stimulation, and inflammation promotion were usually more significant in the low-risk group (Figure 9C). Similar results were observed in the GEO cohort (Figure 9D and 9E).

### Predicting sensitivity to chemotherapy drugs

**Figure 10:**
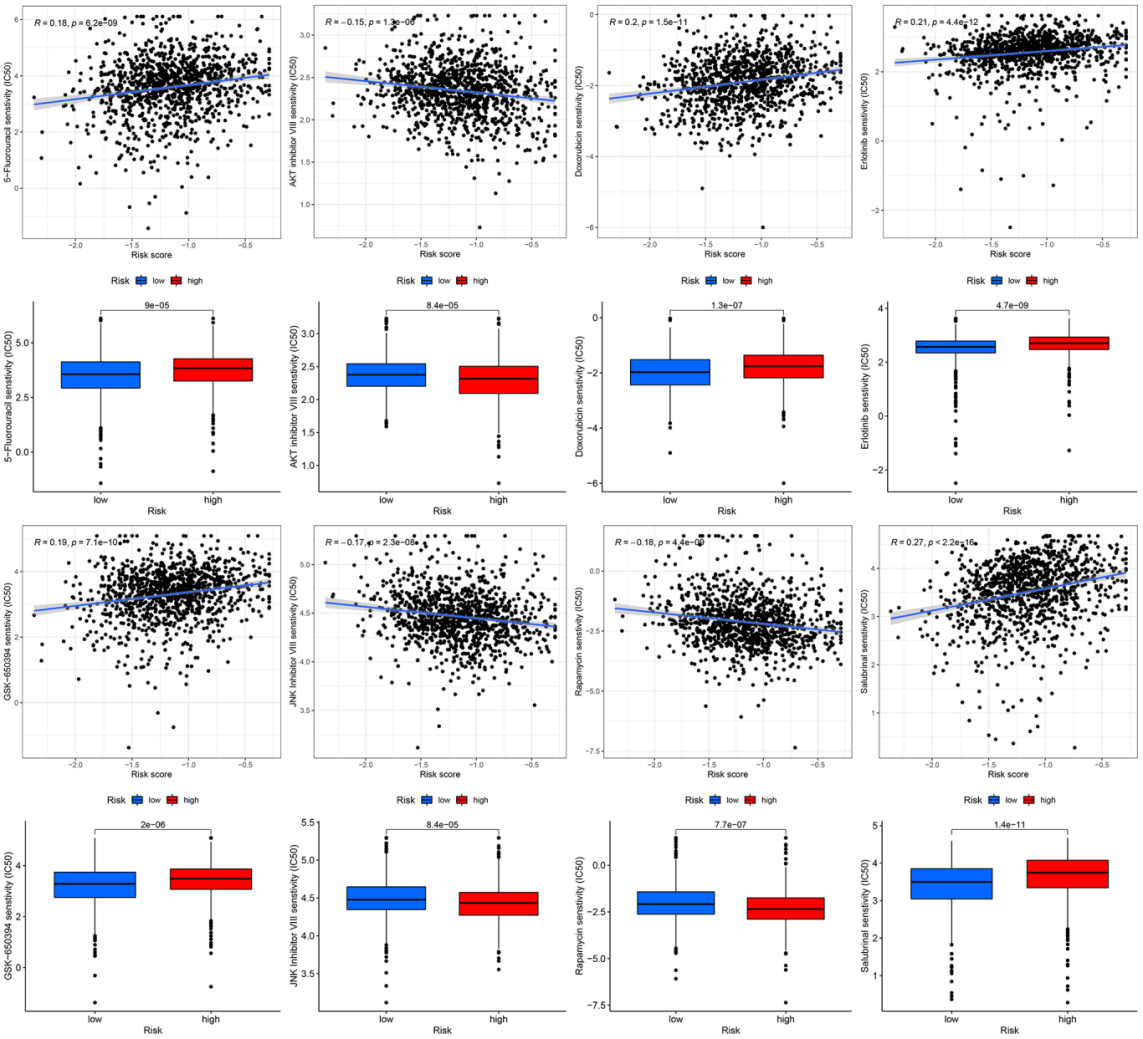
Chemotherapeutic response-prediction of the 13-gene signature.

Currently, chemotherapy remains the mainstay of adjuvant therapy for the treatment of patients with BC [34, 35]. However, many patients are prone to develop resistance to chemotherapy drugs. In the current study, we predicted the response of subgroups to certain chemotherapy drugs (Figure 10). The results revealed that high-risk BC patients showed higher sensitivity to AKT inhibitor VIII, JNK inhibitor VIII, and rapamycin, suggesting that high-risk patients can benefit from therapeutic agents. Additionally, we found that high-risk BC patients had higher estimated IC50s for five chemotherapy drugs (5-Fluorouracil, doxorubicin, erlotinib, GSK-650394, and salubrinal) than that of low-risk BC patients.

## DISCUSSION

Dysregulated gene expression in BC tissues has been extensively investigated. Cancer cells can selectively suppress or increase specific mRNA translation to promote tumor development and metastasis, leading to poor survival of cancer patients [36]. However, there is still no clear understanding of how BC develops and progresses. A new prognostic signature for BC patients, and clarification of potential mechanisms need to be identified. Mitophagy is a type of selective autophagy in which mitochondria are selectively cleared by the autophagy pathway [37, 38]. Mitophagy is critical for intracellular environmental stability, and cells use this mechanism to eliminate mitochondrial dysfunction or reduce the number of mitochondria [39, 40]. Mitophagy can be mediated by a variety of molecular mechanisms such as PINK1 the Parkin, NIX, and FundC1 pathways. Mitophagy disorder leads to a variety of cancers, including rectal cancer, lung cancer, and BC. Deng et al. found that degradation of ULK1 inhibits mitophagy and promotes BC bone metastasis [12]. However, the role of mitophagy in BC has not been fully established. In the present study, we built a 13-gene signature based on DEGs of mitophagy-related tumor classification as a novel biomarker for the prognosis of BC and developed a comprehensive analysis of the signature's value for determining risk stratification, chemotherapy response, and immune activity in patients with BC.

Our 13-gene (ADAM9, MAL2, CLEC3A, TNFRSF14, RELB, SEMA3B, IGFALS, CEBPD, KRTCAP3, CCL19, CHAD, KRT5, and LTF) signature based on DEGs of mitophagy-related tumor classification was conducted for BC. These genes play important roles in various cancers including BC. Xu et al. reported that the circNINL/miR-921 axis could upregulate the expression of ADAM9, the direct target of miR-921, and activate β-catenin signaling to promote the progression of BC [41]. Jun et al. discovered that CLEC3A promotes tumor progression and poor prognosis in BC via the PI3K/AKT signaling pathway [42]. Besides, Fang et al. showed that MAL2 suppresses tumor antigen presentation and drives immune evasion in BC [43]. Additionally, Zhou et al. revealed that ADAM9 may mediate BC progression via AKT/NF-kB signaling [44]. Although these genes are still unknown, we demonstrated their prognostic capacity in BC and confirmed the poor outcome of high-risk BC identified by the signature calculated using gene expression.

The 13 gene signature showed a significant correlation with BC OS when analyzed via univariate Cox. The K–M analysis found that a higher risk score for BC was associated with a poorer prognosis. Meanwhile, our signature was demonstrated to be a reliable prognostic indicator for patients with BC using multivariate Cox regression analysis. ROC curve analysis confirmed the robust predictive capacity of our model. A nomogram was constructed using a 13-gene signature and clinical characteristics. The calibration curves of 1-, 3-, and 5-year survival rates indicated the accuracy of predicting survival probabilities for BC, which illustrated that our nomogram was an excellent predictor. With the development of bioinformatics, accumulating tools have identified various specific genes related to diverse cellular processes. As a disease that is strongly associated with genetic abnormalities, the genome of BC is valuable for exploration. A large body of literature has reported good bioinformatics tools for BC, such as depression-related models, angiogenesis-related models, or lactate metabolism-related models [45–47]. However, no mitophagy-related signature of BC has been studied, which in our study showed excellent prognostic ability and was related to distinct immune cell infiltration patterns.

Next, we performed a functional analysis of DEGs between the high- and low-risk subtypes to explore the putative pathways and functions of the 13-gene signature. GO enrichment revealed these DEGs were related with “chemokine-mediated signaling pathway” and “response to chemokine”. Chemokines are signal proteins or small cytokines that enhance the antitumor immune response by recruiting nearby immune cells [48]. For example, B cells can be activated by CXCR5, and T and NK cells can be induced to be enriched in tumors by CXCL9/10/11 [49]. Previous evidence has confirmed the vital roles of chemokines in the progression of BC, such as the overexpression of chemokines in mammary fibroblasts regulated by MEKK1, which can form a TME that supports the migration of BC cells [50], as well as the promotion of migration of ING4-deficient BC cells induced by CXCL10 chemokines [51]. Furthermore, previous studies have reported that chemokines are closely related to M1/M2 polarization, which can further promote tumor development [52]. Our GO analysis revealed a possible connection between chemokines, TME, and BC progression.

KEGG enrichment showed significant enrichment of “viral protein interaction with cytokine and cytokine receptor” and “NF-kappa B signaling pathway”. Research of M.SR et al. suggests NF-kB plays an essential role in antitumor immunity. NF-kB is important for the formation of B lymphoid tissues and for the differentiation and maturation of B cells. Malfunctioning of NF-kB may decrease immunogenicity owing to the multifaceted role of this transcription factor in immunity [53]. Abundant evidence has demonstrated the positive functions of the NF-kB signaling pathway in promoting BC progression. Interestingly, Yi et al. reported that lncRNA lnc-SLC4A1-1 can induce BC development by activating the CXCL8 and NF-kB signaling pathways [54]. Combined with our functional enrichment analysis, we propose that there must be some crosstalk between chemokines, the NF-kB signaling pathway, and BC. Subsequently, we explored whether there was a connection between mitophagy, NF-kB, and BC. However, this connection has not yet been explored but we obtained some interesting findings. Zhao et al. reported that metformin could rescue mitophagy in high glucose-challenged human renal epithelial cells by downregulating the NF-kB signaling pathway [55]. Further studies will be conducted to address these questions. In addition, the GSEA results showed that “cell cycle” and “homologous recombination” were enriched pathways in the high-risk group. In the low-risk group, the top 5 enriched pathways were “cytokine-cytokine receptor interaction,” “neuroactive ligand receptor interaction,” “hematopoietic cell lineage,” “chemokine signaling pathway” and “primary immunodeficiency”. These results suggest that the 13 genes might influence the tumorigenesis and progression of BC through immune and tumor-related signaling pathways.

TME plays a vital role in BC immunotherapy [56, 57]. Analysis of TME may help us to better understand how mitophagy influences the outcomes of patients with BC. Therefore, we assessed the proportion of various immune cells in BC using six commonly used algorithms. In low-risk patients, the TME was significantly infiltrated by B cells, DC, iDC, CD4+T cells, mast cells, CD8+T cells, neutrophils, pDC, T helper cells, Tfh, and TIL. These immune cells can affect BC development by regulating the antitumor immune response. Further studies revealed that CCR, MHC class I, checkpoint, cytolytic activity, HLA, parainflammation, T cell co-stimulation, inflammation promotion, and type II IFN response were enriched in the low-risk group. In addition, we evaluated the effectiveness of certain chemotherapies for different subtypes of BC. The results revealed that low-risk patients had lower estimated IC50s for five chemotherapy drugs (5-Fluorouracil, doxorubicin, erlotinib, GSK-650394, and salbrinal) than that of high-risk patients. Moreover, high-risk patients were more sensitive to AKT inhibitor VIII, JNK inhibitor VIII, and rapamycin, suggesting that high-risk patients can benefit from these chemotherapeutic agents. These results have the potential to guide therapy selection for each patient with BC.

Our study provides an exhaustive summary of all the possible mechanisms and gene alterations of the 13-gene signature based on DEGs of mitophagy-related tumor classification in BC, provides a solid foundation for future research, and can guide prognostic biomarkers of therapeutic strategies for patients with BC.

## Supplementary Materials

Supplementary Table 1
