# Supplementary material for: A signature constructed with mitophagy-related genes to predict the prognosis and therapy response for breast cancer
Source: Aging (Albany NY). 2022 Aug 5;14(15):6169–86. doi: 10.18632/aging.204209 (PMC9417220; doi:10.18632/aging.204209)
Supplement: Supplementary Table 1 [file aging-14-204209-s001.pdf]

SUPPLEMENTARY TABLE

Supplementary Table 1. Mitophagy-related genes.

ATG12
ATG5
CSNK2A1
CSNK2A2
CSNK2B
FUNDC1
MAP1LC3A
MAP1LC3B
MFN1
MFN2
MTERF3
PGAM5
PINK1
PRKN
RPS27A
SQSTM1
SRC
TOMM20
TOMM22
TOMM40
TOMM5
TOMM6
TOMM7
TOMM70
UBA52
UBB
UBC
ULK1
VDAC1
